# Early New Ischemic Lesions Located Outside the Initially Affected Vascular Territory Appear More Often in Stroke Patients with Elevated Glycated Hemoglobin (HbA1c)

**DOI:** 10.3389/fneur.2017.00606

**Published:** 2017-11-14

**Authors:** Tim Bastian Braemswig, Christian H. Nolte, Jochen B. Fiebach, Tatiana Usnich

**Affiliations:** ^1^Department of Neurology, Charité – Universitätsmedizin Berlin, Corporate Member of Freie Universität Berlin, Humboldt-Universität zu Berlin, Berlin Institute of Health, Berlin, Germany; ^2^Berlin Institute of Health (BIH), Berlin, Germany; ^3^Center for Stroke Research Berlin (CSB), Charité—Universitätsmedizin Berlin, Berlin, Germany

**Keywords:** stroke, diabetes mellitus, hemoglobin A, glycosylated, magnetic resonance imaging, new ischemic lesions, diffusion magnetic resonance imaging

## Abstract

**Background:**

Early new ischemic lesions are common in patients with an acute ischemic stroke. These new ischemic lesions may represent the natural course of the initial stroke or *de novo* events.

**Objective:**

We hypothesized that early new ischemic lesions located outside the initially affected vascular territory would point at *de novo* events. Therefore, we differentiated new ischemic lesions located outside the initially affected vascular territory from those occurring only inside the initially affected vascular territory to identify risk factors that are associated with *de novo* events.

**Methods:**

Stroke patients underwent three magnetic resonance imaging examinations (at 3-T): on admission, on the next day and 4–7 days after symptom onset (clinicaltrials.gov: NCT00715533). Diffusion-weighted imaging (DWI) lesions were delineated, coregistered, and then analyzed for new hyperintensities on follow-up examinations by raters blinded to clinical details. Patients were classified as having “new distant lesions” if new DWI lesions appeared outside or both outside and inside the initially affected vascular territory or “new local lesions” if they were only inside.

**Results:**

115 patients with early new DWI lesions constitute the study population. Sixteen patients (14%) had new distant lesions and 99 patients (86%) had new local lesions. In comparison between patients with new distant and new local lesions, patients with new distant lesions had significantly more often elevated glycated hemoglobin (HbA1c ≥ 6.5%; *p* = 0.022).

**Conclusion:**

Our data indicate that patients with elevated HbA1c have an increased risk for new, *de novo* ischemic lesions in the acute phase after an ischemic stroke.

## Introduction

Early new ischemic lesions are common in the acute phase after an ischemic stroke. Studies using serial magnetic resonance imaging (MRI) within 1 week after an initial ischemic stroke found that new lesions on diffusion-weighted imaging (DWI) appear in >30% of all patients. Most of the patients with these new ischemic lesions lack new clinically overt symptoms (silent strokes) (1–5). However, stroke patients with early new ischemic lesions are at an increased risk for subsequent clinically overt ischemic events (6).

Early new ischemic lesions in the acute phase after an ischemic stroke may represent different pathophysiological entities. Previously, it has been suggested that in patients with a (spontaneous or post-thrombolytic) vessel recanalization, new lesions may result from incomplete clot dissolution and distal embolization. These new lesions are directly related to the initial ischemic event and therefore represent the natural course of the disease (2–4, 7). By contrast, the occurrence of new lesions located outside the initially affected vascular territory cannot be explained by incomplete clot dissolution of the embolus. Therefore, we hypothesized that these new lesions represent *de novo* events (possibly originating from a proximal source such as the aortic arch or heart).

In this study, we investigated factors associated with the occurrence of new DWI lesions in relation to the initially affected vascular territory [defined as the territory of the left internal carotid artery (anterior left), the right internal carotid artery (anterior right), and the vertebrobasilar circulation (posterior) (8)] to identify risk factors that are associated with *de novo* events.

## Materials and Methods

### Patients

Patients were included in this study as part of an observational study (1000Plus) conducted by the Center for Stroke Research Berlin (CSB) at the Campus Benjamin Franklin of the Charité—Universitätsmedizin Berlin (clinicaltrials.gov: NCT00715533). The study enrolled all patients with an acute ischemic stroke within the last 24 h who were eligible for MRI. Details of this study have been previously published (2–4, 9). Patients recruited between March 2008 and December 2010, with a complete set of three examinations within the first week after symptom onset and initial DWI lesion(s) were included in the analysis of this study. Patients with ischemic lesions in all three vascular territories (anterior left, anterior right, and posterior) on first MRI examination were excluded from the analysis, as were patients who underwent endovascular interventions. All patients received standard stroke unit care. The study was approved by the local Ethics Committee of the Charité—Universitätsmedizin Berlin (EA4/026/08) and all patients gave written informed consent (2–4).

### Imaging Protocol

The examinations were performed on a 3-T MRI scanner (Tim Trio, Siemens Medical, Erlangen, Germany). DWI was acquired with 230 mm field-of-view, fifty 2.5 mm axial slices without extra gap, with 1 *b* = 0 and along 6 directions of *b* = 1,000 s/mm^2^, repetition time/echo time = 7,600/93 ms, and an acquisition matrix of 192 × 192. Other sequences included axial fluid-attenuated inversion recovery, T2*-weighted imaging, perfusion imaging, and three-dimensional time-of-flight MR angiography of the intracranial circulation. We conducted three MR examinations: on admission, on the following day, and 4–7 days after onset of symptoms (2–4, 9).

### Image Analysis

DWI were pseudonymized and afterwards reviewed in random order by raters blinded to clinical information. Hyperintensities on initial DWI were delineated manually, coregistered to the DWI on second day, and resliced to 1 mm isotropic voxel size. Coregistered DWIs were analyzed visually for new hyperintensities separate from the index lesion through slice-by-slice comparison of the first and second, as well as the second and third DWI. In this study, all new diffusion hyperintensities regardless of size and ADC value were considered.

**Figure 1:**
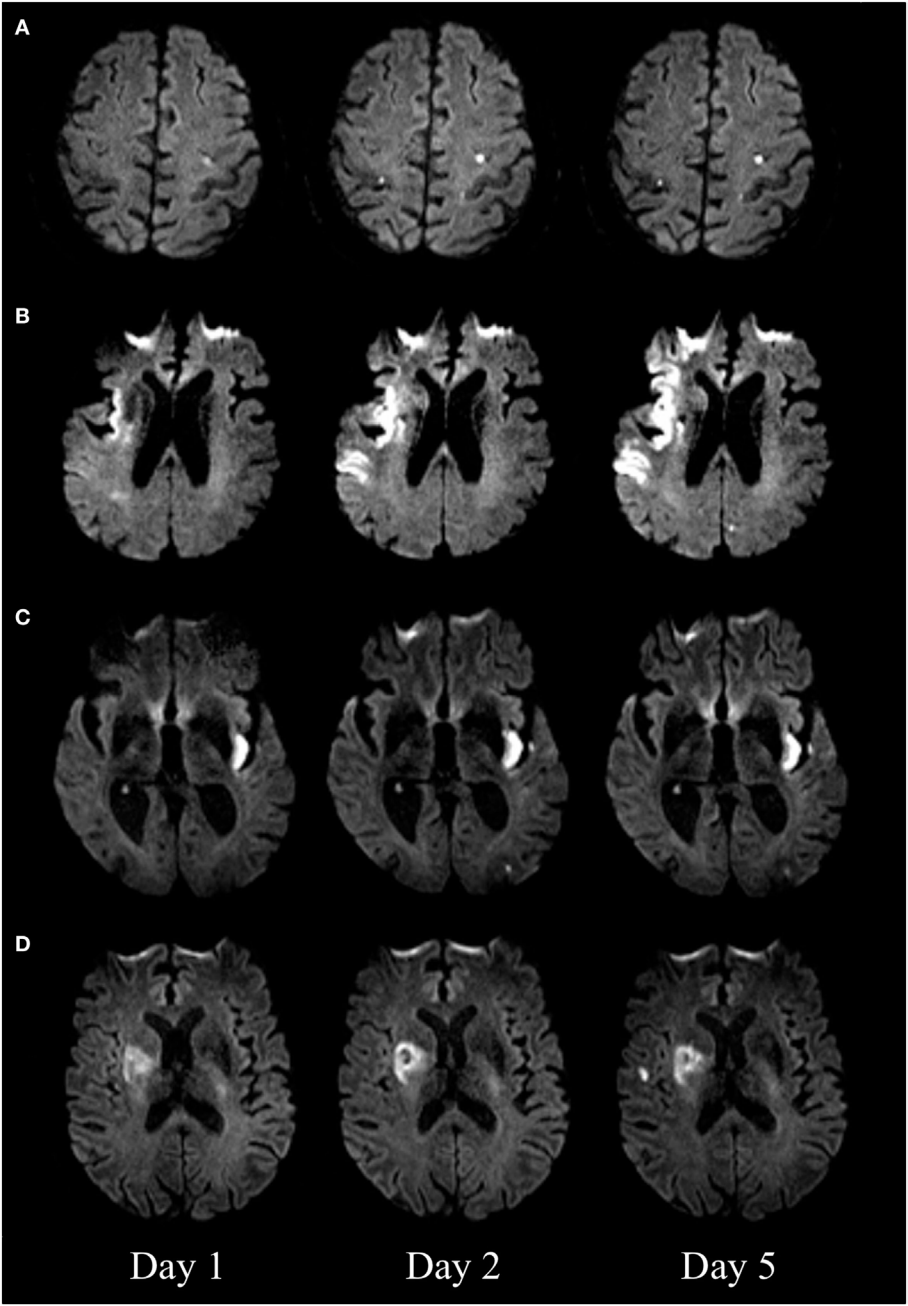
Diffusion-weighted imaging (DWI) of four patients with new DWI lesions. New distant lesions, appearing on day 2 **(A)** and day 5 **(B)**. New local lesions appearing on day 2 **(C)** and day 5 **(D)**.

The initial pattern of infarction was assessed on the first DWI. We defined three vascular territories: the territory of the left internal carotid artery (anterior left), the right internal carotid artery (anterior right), and the vertebrobasilar circulation (posterior) (8). The fetal type of posterior cerebral artery was taken into consideration. New DWI lesions were classified as being “new distant lesions” if they were either outside or both outside and inside the initially affected vascular territory. New DWI lesions were classified as “new local lesions” if they were inside the initially affected vascular territory (Figure 1).

### Clinical Data

Sociodemographic and laboratory data were collected from the medical records. Patients with a known medical history of diabetes mellitus or patients with pre-existing antidiabetic medication were defined as having a “history of diabetes mellitus.” In addition, we surveyed glycated hemoglobin (HbA1c; method: high performance liquid chromatography) in all patients on admission and used an HbA1c cut point of ≥6.5% as a diagnostic criteria for diabetes mellitus according to international guidelines (10). Symptomatic carotid stenosis was defined as stenosis >50% on color-coded duplex sonography or magnetic resonance angiography that explained any symptomatic lesion. Stroke subtype was classified according to Trial of Org 10172 in Acute Stroke Treatment criteria after a review of the clinical and imaging information (11). All patients were assessed for stroke severity directly before the first MR examination and daily until the day of discharge by physicians certified to assess the National Institutes of Health Stroke Scale (NIHSS) (12). Secondary prevention measures were implemented according to current practice guidelines (13). All patients were assessed four times daily for new neurological symptoms by a stroke physician dedicated to stroke care and patients’ complaints were taken into account. At the time of follow-up clinical examination, physicians were blinded to MRI results. A clinical stroke recurrence was defined as a functional deterioration in the neurological status clearly distinct from that of the index stroke, attributable to a different vascular territory or the new event was of a different stroke subtype (1, 2, 4).

### Statistics

Clinical and neuroradiological characteristics of patients were compared using chi-square test for nominal and categorical variables. Statistical significance was determined at an alpha level of 0.05 (2–4). Statistical analyses were performed using SPSS 22.

## Results

Out of 340 patients examined, 130 patients had new DWI lesions. Fifteen patients had ischemic lesions in all three vascular territories on first MRI examination and were excluded from this analysis. Therefore, 115 patients with new DWI lesions constitute the (main) study population [37% female, median age 72 years (IQR 66–79), median NIHSS 4 (IQR 2–8)]. Of these 115 patients, clinical stroke recurrence occurred in three patients within the first week (3%). Sixteen patients (14%) had new DWI lesions outside (or both outside and inside) the initially affected vascular territory (new distant lesions) and 99 patients (86%) had new DWI lesions inside the initially affected vascular territory (new local lesions). Patients with new distant lesions had significantly more often elevated glycated hemoglobin (HbA1c ≥ 6.5%, *p* = 0.022; Table 1).

**Table 1:** Sociodemographic and clinical parameters: patients with new lesions inside and outside the initially effected vascular territory.

Characteristics	New local lesions (*n* = 99)	New distant lesions (*n* = 16)	*p*
Sex (women)	38 (38.4%)	5 (31.3%)	0.584
Age (>70 years)	58 (58.6%)	11 (68.8%)	0.441
NIHSS (>3)	52 (52.5%)	9 (43.8%)	0.515
Hyperlipoproteinemia	45 (45.5%)	11 (68.8%)	0.084
History of diabetes mellitus	22 (22.2%)	6 (37.5%)	0.186
Arterial hypertension	80 (80.8%)	14 (87.5%)	0.520
Atrial fibrillation	36 (36.4%)	3 (18.8%)	0.167

TOAST			0.657
– LAA	29 (29.3%)	6 (37.5%)	
– CE	37 (37.4%)	4 (25%)	
– Small vessel	0 (0%)	0 (0%)	
– Other determined	3 (3.0%)	0 (0%)	
– Undetermined	30 (30.3%)	6 (37.5%)	

CHD	15 (15.2%)	2 (12.2%)	0.782
PAOD (95/16 pat.)	9 (9.5%)	2 (12.5%)	0.708
Previous stroke	25 (25.3%)	3 (18.8%)	0.574
Symptomatic carotid stenosis	11 (11.1%)	2 (12.5%)	0.871
Thrombolysis	38 (38.4%)	5 (31.3%)	0.584
Vessel recanalization	34 (34.3%)	7 (43.8%)	0.466
Antiplated therapy within 48 h after stroke	94 (94.9%)	15 (93.8%)	0.841

Glucose (mmol/l) (90/16 pat.)			
– 10	12 (13.3%)	1 (6.3%)	0.462
– >7	39 (43.3%)	7 (43.8%)	0.975

LDL (>3.4 mmol/l) (90/15 pat.)	30 (33.3%)	5 (33.3%)	1.000

INR (96/16 pat.)			
– 2	4 (4.2%)	1 (6.3%)	0.709
– >1.7	4 (4.2%)	1 (6.3%)	0.709

HbA1c (≥6.5%) (90/14 pat.)	15 (16.5%)	6 (42.9%)	0.022

*NIHSS, National Institutes of Health Stroke Scale; CHD, coronary heart disease; PAOD, peripheral artery occlusive disease; LDL, low density lipoprotein; INR, international normalized ratio; TOAST, Trial of Org 10172 in Acute Stroke Treatment*.

In a further analysis, comparison between patients with new distant lesions (*n* = 16) and all other acute stroke patients (both with new local lesions and without new lesions; *n* = 324) confirmed that new distant lesions appear significantly more often in patients with elevated glycated hemoglobin (42.9 vs. 18.7%, *p* = 0.027; data were available in 14/16 and 283/324 patients, respectively).

Out of 28 patients with a “history of diabetes mellitus,” 27 patients (96%) had diabetes mellitus type 2. In patients with new distant lesions, two patients had a medical intervention to lower blood glucose and one patient had a medical intervention to lower blood pressure during the course of the study. No patient with new distant lesions had hypoglycemia during the course of the study (data were available in 14/16 patients).

## Discussion

For the first time, we found a significant association between elevated glycated hemoglobin (HbA1c ≥ 6.5%) and early new ischemic lesions located outside the initially affected vascular territory in acute stroke patients. New ischemic lesions in the acute phase after stroke may represent different pathophysiological entities, namely either the natural course of the initial stroke or *de novo* events (3, 5, 7). We hypothesized that early new ischemic lesions located outside the initially affected vascular territory could be interpreted as *de novo* events. Previously, it was reported that new ischemic lesions after stroke are associated with subsequent clinically overt ischemic events (1–4, 6). Assuming that new ischemic lesions represent different pathophysiological entities, especially those patients with new, *de novo* ischemic lesions might be at an increased risk for subsequent clinically manifest strokes.

We found a significant association between early new ischemic lesions located outside the initially affected vascular territory (new distant lesions) and elevated HbA1c. Elevated HbA1c was found to be an independent predictor for worse neurological and functional outcome after stroke and to predict future ischemic strokes (14–16). Poor glycemic control leads to endothelial dysfunction, coagulative activation, and platelet hyper-reactivity (17). Our data indicate that patients with elevated HbA1c also have an increased risk for new, *de novo* ischemic lesions in the acute phase after an ischemic stroke.

Interestingly, new distant lesions did not appear significantly more often in patients with a known medical history of diabetes mellitus or in patients with pre-existing antidiabetic medication (“history of diabetes mellitus”), which may implicate that elevated HbA1c is more sensitive in detecting poor glycemic control than surveying the patients’ past medical history.

It might come as a surprise that there was no significant association between new distant lesions and atrial fibrillation. The time interval investigated in our study was 1 week only and studies on (clinically manifest) stroke recurrence typically report on considerably longer time intervals up to several years (18). New cardiac thrombus formation may not happen within such a short-time interval.

Limitations of this study have to be considered. First, this is a single-center *post hoc* analysis. Second, the number of patients with new distant lesions was too small for multiple testing. Therefore, we cannot exclude false positive results. Third, due to the single-center setup, results cannot be generalized easily. Fourth, because of ethical considerations, patients were only included in the absence of contraindications to MRI and after giving written informed consent, which entails selection bias to the disadvantage of more severely affected and aphasic patients.

In conclusion, elevated HbA1c (≥6.5%) is associated with new, *de novo* ischemic lesions in patients with an acute ischemic stroke. Future prospective studies with long-term follow-up of stroke patients with elevated HbA1c and new ischemic lesions are necessary to confirm this novel finding.

## Ethics Statement

The study was approved by the local Ethics Committee of the Charité—Universitätsmedizin Berlin (EA4/026/08) and all patients gave written informed consent.

## Author Contributions

TBB: data collection, data analysis and interpretation, drafting the article, and final approval. CHN: conception/design of the work, data analysis and interpretation, critical review, and final approval. JBF: data collection, critical review, and final approval. TU: conception/design of the work, data collection, data analysis and interpretation, drafting the article, and final approval.

## Conflict of Interest Statement

The authors declare that the research was conducted in the absence of any commercial or financial relationships that could be construed as a potential conflict of interest.
